# Dynamic control and manipulation of near-fields using direct feedback

**DOI:** 10.1038/s41377-024-01610-2

**Published:** 2024-10-24

**Authors:** Jacob Kher-Aldeen, Kobi Cohen, Stav Lotan, Kobi Frischwasser, Bergin Gjonaj, Shai Tsesses, Guy Bartal

**Affiliations:** 1 The Andrew & Erna Viterbi Faculty of Electrical & Computer Engineering, Technion—Israel Institute of Technology, Haifa, 3200003 Israel; 2 Department of Physical Engineering, Polytechnic University of Tirana—Faculty of Physical & Math Engineering, Tirana, 1000 Albania; 3 Faculty of Medical Sciences, Albanian University, Durrës Street, Tirana, 1000 Albania; 4 Department of Physics, MIT—Harvard Center for Ultracold Atoms and Research Laboratory of Electronics, Massachusetts Institute of Technology, Cambridge, MA USA

**Keywords:** Nanophotonics and plasmonics, Nonlinear optics, Sub-wavelength optics

## Abstract

Shaping and controlling electromagnetic fields at the nanoscale is vital for advancing efficient and compact devices used in optical communications, sensing and metrology, as well as for the exploration of fundamental properties of light-matter interaction and optical nonlinearity. Real-time feedback for active control over light can provide a significant advantage in these endeavors, compensating for ever-changing experimental conditions and inherent or accumulated device flaws. Scanning nearfield microscopy, being slow in essence, cannot provide such a real-time feedback that was thus far possible only by scattering-based microscopy. Here, we present active control over nanophotonic near-fields with direct feedback facilitated by real-time near-field imaging. We use far-field wavefront shaping to control nanophotonic patterns in surface waves, demonstrating translation and splitting of near-field focal spots at nanometer-scale precision, active toggling of different near-field angular momenta and correction of patterns damaged by structural defects using feedback enabled by the real-time operation. The ability to simultaneously shape and observe nanophotonic fields can significantly impact various applications such as nanoscale optical manipulation, optical addressing of integrated quantum emitters and near-field adaptive optics.

## Introduction

The ability to shape and control light is of fundamental importance in a wide range of fields, including imaging and spectroscopy^1–5^, optical trapping^6–8^, quantum photonics^9^, optical communication^10^, nonlinear optics^11^ and more. While the early stage of light shaping was within the framework of adaptive optics^12,13^, contemporary methods to control light include the spatial light modulator (SLM)^14–17^ capable of controlling the phase and amplitude of optical waves at high spatial resolution and with a large dynamic range.

The importance of optical wave-front shaping is evident in many fields, such as trapping and manipulation of nanoparticles via holographic optical tweezers, which finds widespread applications in biology, biomedical and physical sciences^6^. It has also been instrumental in demonstrating the ability to focus light through opaque media^14–16^, thereby opening up new possibilities for non-invasive imaging^2,3,18^.

The potential use of wavefront shaping in nanophotonics can provide the control of light at subwavelength scales, making a profound impact in numerous applications; it can divide power among nano-scale focal spots in controlled manner to improve super-resolution imaging and metrology^19,20^; as well as enhance light-matter interactions by accurately addressing nano-emitters or rotate particles^21,22^.

Such shaping, however, becomes limited at the absence of direct feedback as the coupling mechanism to nanophotonic modes, utilizing e.g. a grating, does not preserve the wavefront of the incident beam. Near-field imaging approaches which are based on raster scanning, post processing or large integration times^23–25^ are able to monitor near-fields shaped either by the polarization of the incident beam^26–32^ or by SLM^33^ but cannot provide real-time monitoring for, e.g. tracking changes in the near-fields or fix broken or distorted wavefronts. A direct feedback could only be provided by scatterers that perturb the field^19,20,34,35^, limiting the applicability of nano-scale wavefront shaping in dynamic processes.

A recent advance, capable of direct imaging of nanophotonic fields^36^ can provide instantaneous and non-perturbating attributes, thereby enabling the desired real-time feedback.

Here, we demonstrate active control over nanophotonic fields with real-time feedback. Our methodology involves the generation of shaped surface plasmon polaritons (SPPs), actively controlled by an SLM and monitored in real-time. This dynamic control encompasses various capabilities, including translating and splitting a plasmonic focal spot over several microns with 30 nm precision and dynamic switching of the angular momentum of near-field modes. We further demonstrate how this capability allows to fix and correct nanophotonic patterns corrupted by structural defects. Our real-time mapping technique, providing the real-time feedback mechanism, exploits the 3rd-order nonlinearity of metal using surface plasmon polaritons (SPPs). However, it is inherently present in metals, semiconductors and interfaces in general and was already proven successful in Silicon Photonics^37^ and phonon-polariton in Silicon Carbide^38^.

## Results

**Fig. 1:**
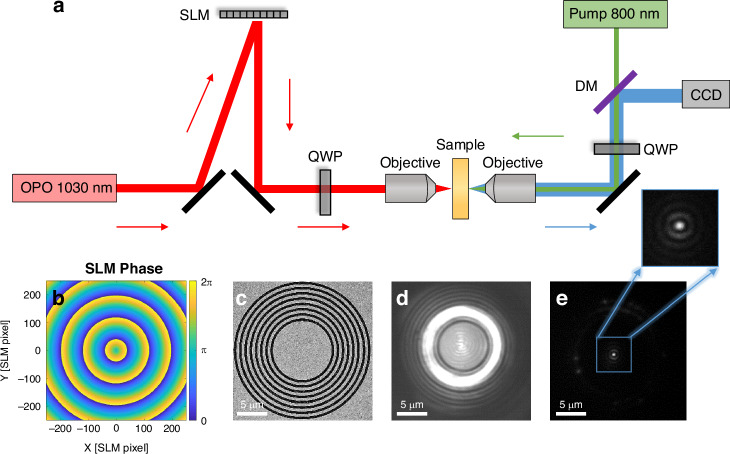
Concept and system. **a** Illustration of the Nonlinear Nearfield Optical Microscope (NNOM) used for mapping wavefront-controlled near-field patterns. SLM – Spatial light modulator. OPO – Optical parametric oscillator. DM – dichroic mirror. QWP – Quarter waveplate. The objective used to focus the OPO signal on the sample has NA of 0.42 and magnification of x 20, while the objective used for imaging has NA of 0.9 and magnification of x 100. **b** Linearly-ascending radially symmetric phase mask stored on the SLM for producing a ring-shape beam. **c** SEM image of the sample: a circularly symmetric grating carved in a 180 nm thick gold layer. **d** Optical image showing the grating illuminated by the equal-phase ring at 1030 nm wavelength. **e** Real-time mapping of the excited near-field pattern at 667 nm using NNOM. The inset shows a zoom-in on the resultant 0^th^ order Bessel mode

The experimental apparatus is described in Fig. 1a. We generate the nanophotonic patterns on the gold-air interface using 140 fs pulsed, circularly polarized laser at a wavelength of 1030 nm. The laser beam is shaped using a phase-only SLM, positioned in the Fourier plane of the metallic layer. The SLM imprints a radially-ascending phase on the light reflected off of it, as illustrated in Fig. 1b. This phase modulation results in the creation of a ring-shaped beam which impinges the coupling grating, carved in the metallic layer, as shown in Fig. 1c. The diameter of this ring-shaped beam can be precisely adjusted to match the size of the grating (Fig. 1d), which improves the coupling efficiency and reduces heating of the metallic layer. A second pulsed laser beam (‘pump’), operating at a wavelength of 800 nm and phase-locked with the first beam generates the nonlinear beam, at 667 nm wavelength, which contains the information on the near-field signal^36^. The pump beam is focused to a 4 μm diameter focal spot and is circularly polarized such that the nonlinear interaction produces the spatial information encoded within the field component of the SPP mode that rotates at the same direction^27,36^. We use 100 mW average power corresponding to power density of 16 mWatt/μm^2^. we find experimentally that the damage threshold intensity is ~30 mWatt/μm^2^. Figure 1e depicts the resultant near-field pattern as recorded on the CCD camera using the aforementioned nonlinear process.

The SPP pattern is generated by illuminating a phase-encoded ring generated in the Fourier plane of the SLM. A radially-symmetric, radially-ascending phase is stored on the SLM, such that the electric field of a Gaussian beam reflected from the SLM is $E\left(r,\theta \right)={e}^{-\pi {(\frac{r}{{r}_{0}})}^{2}}{e}^{{jar}}$, where ${r}_{0}$ represents the size of the Gaussian beam and $a$ is the radial rate of the phase advance.

The field on the grating is, therefore, the Fourier transform of the beam reflected from the SLM,

$${\mathcal{F}}\left[E\left(r,\theta \right)\right]=\frac{{{r}_{0}}^{2}}{4\pi }{e}^{-\frac{1}{4\pi }{\left({k}_{r}{r}_{0}\right)}^{2}}* \delta \left({k}_{r}-a\right)=\frac{{{r}_{0}}^{2}}{4\pi }{e}^{-\frac{1}{4\pi }{\left({k}_{r}-a\right)}^{2}{{r}_{0}}^{2}}$$

Where the ring diameter is determined by $a$. The surface wave generated by the ring-shaped beam incident on a circular grating coupler can be calculated using a Huygens-principle simulation^39^ (see supplementary for more details). The field components can be represented as two rotating in-plane components and one out-of-plane component^36^:

$$\left(\begin{array}{c}{E}_{{{\sigma }}_{-}}^{{SPP}}\\ {E}_{{{\sigma }}_{+}}^{{SPP}}\\ {E}_{z}^{{SPP}}\end{array}\right)=\left(\begin{array}{c}\frac{{E}_{x}^{{SPP}}+i{E}_{y}^{{SPP}}}{\sqrt{2}}\\ \frac{{E}_{x}^{{SPP}}-i{E}_{y}^{{SPP}}}{\sqrt{2}}\\ {E}_{z}^{{SPP}}\end{array}\right)\propto \left(\begin{array}{c}{J}_{2}\left({k}_{{SPP}}{\rho }\right){e}^{i2\theta }\\ {J}_{0}\left({k}_{{SPP}}{\rho }\right)\\ {J}_{1}\left({k}_{{SPP}}{\rho }\right){e}^{i\theta }\end{array}\right){e}^{-\left|{k}_{z}\right|z}$$

Where the in-plane field is expressed via its rotating field components ${E}_{{\sigma }_{+}}^{{SPP}}={E}_{x}^{{SPP}}+j{E}_{y}^{{SPP}}$ and ${E}_{{\sigma }_{-}}^{{SPP}}={E}_{x}^{{SPP}}-j{E}_{y}^{{SPP}}$^36^. By applying a radially-ascending phase with no azimuthal twist, this SPP field component takes the form of a 0th order Bessel mode. Illuminating the sample with a ${\hat{\sigma }}_{+}$ polarized pump recovers the shape of the ${\hat{\sigma }}_{+}$ component of the plasmonic vector field at the interface, i.e., ${J}_{0}\left({k}_{{SPP}}\rho \right)$, as shown in Fig. 1e, constituting a plasmonic focal spot.

**Fig. 2:**
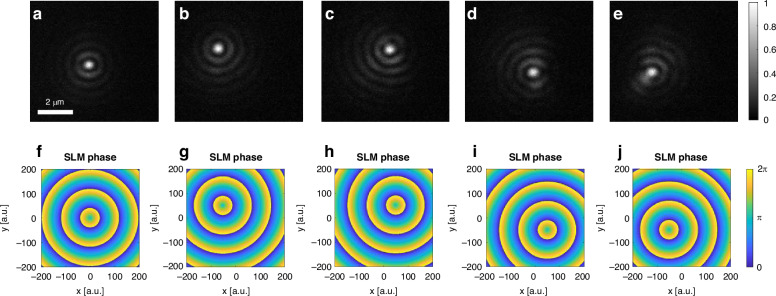
Control over a plasmonic focus. Different positions of the plasmonic focus inside an area of 1.5 µm x 1.5 µm. The plasmonic focus is moving from the center (**a**) to (-0.75,0.75) µm (**b**) and continue to the other side points in (**c**–**e**). **f**–**j** The SLM phase masks desired for the measurements (**a**–**e**) respectively

A prominent implication of the new ability shown herein is a swift control over the focal spot that can be achieved by merely shifting the phase pattern on the SLM. Such a shift results in a linear phase gradient imprinted on the ring incident on the sample which, in turn, generates a constructive interference of SPPs at a location corresponding to the shift of the pattern on the SLM. Figure 2 portrays the full control over the position of the nanoscale SPP focal spot within an area spanning several square microns. Five different locations of the SPP focal spot, each separated by a distance of 1.5 µm, are shown with little to no degradation. The supplementary movie S1 shows the real-time observation of such translation, where the plasmonic focal spot is translated over different positions along a square trajectory, more details and simulations are provided in the supplementary section. The precision on the focal spot location is dictated by the number of pixels used in the SLM. In the current optical setup, a translation of the focal spot by a distance of 1.5 µm requires a shift of 50 pixels on the SLM. Furthermore, the real-time mapping of the near-field allows to monitor and record a pixel-by-pixel translation of a single SPP focal spot in real-time, as shown in supplementary movie S2. While the spot size is limited by the plasmon wavelength, the translation precision is restricted by the optical system and the SLM pixel density, making it 30 nm in this system. The detection resolution, however, is limited by the magnification of the optical system and the pixel density of the camera which makes it 60 nm as can be observed in movie S2.

The spot size itself can also be decreased by increasing its spatial frequencies range. This was achieved through various methods, such as utilizing short-range plasmonic modes^28,40,41^ or periodic nanostructure^42^. The combination of this tight focusing ability with precision tuning could impact various fields related to nanophotonics such as near-field adaptive optics and plasmonic microscopy^20^, trapping and manipulation of nanoparticles in plasmonic tweezers^43,44^ and many others.

**Fig. 3:**
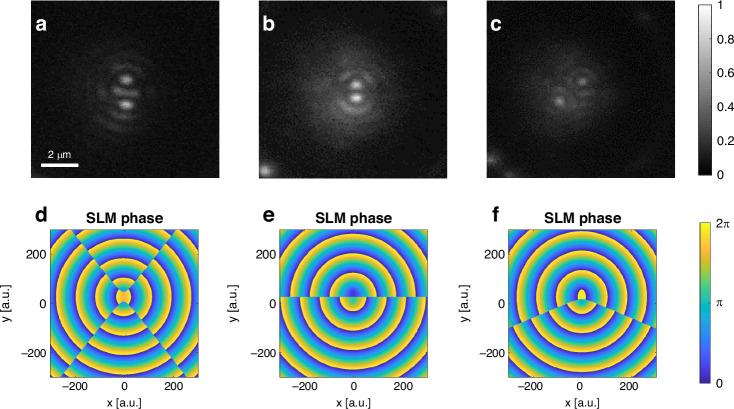
Simultaneous manipulation of different plasmonic foci. **a** A pair of plasmonic foci can be achieved via splitting a plasmonic focal spot by adding together two shifted radial phases on the SLM, each corresponding to a shifted plasmonic focus. **b**-**c** the two emerging plasmonic foci can be manipulated and rotated simultaneously by varying the locations and relative phases of the shifted radial patterns. **d**–**f** The SLM phase masks desired for the measurements (**a**–**c**) respectively

Notwithstanding the importance of controlling the position of a single nanophotonic focal spot, some applications benefit control and manipulation of multiple foci on a surface. Dynamic optical trapping of multiple particles simultaneously, for example, is often used for DNA unfolding^6,43–45^ and performing it on-chip at nanoscale control can open new avenues for these applications. We show here a major step towards this goal, achieved by superimposing translated phase patterns on the SLM, resulting in creation of pairs of focal spots that can be individually manipulated and rotated. Figure 3 and supplementary movie S3 depict the generation of such a pair of focal spots and their manipulation in terms of separation and rotation.

The ability to simultaneously shape and map the near-field opens new degrees of freedom such as toggling between multiple angular momenta of the near-field. Controlling the orbital angular momentum (OAM) of the near-field can be done by imprinting an azimuthally-ascending phase gradient on the SLM, resulting in a winding number $q$, corresponding to the number of times the azimuthal phase completes integer cycles of 2π (see supplementary for more details). The resultant wavefront takes on a spiral form such that the near-field pattern acquires a topological charge of the same order, manifesting as an m^th^ order near-field Bessel mode.

**Fig. 4:**
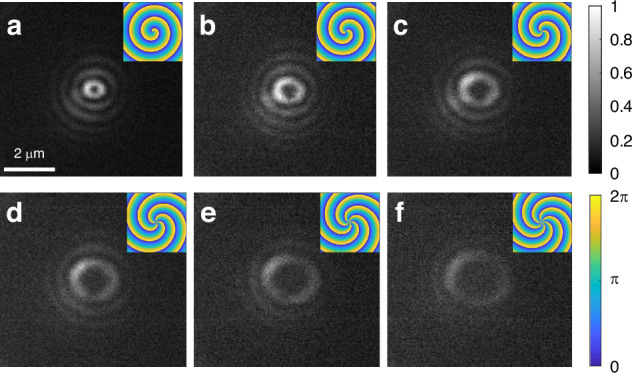
Controlling near-field angular momentum by shaping the far-field phase. **a**–**f** Near-field Bessel modes of order 1–6, respectively, all obtained using similar experimental conditions, i.e., the same coupling grating and polarization state. Insets show the corresponding phase patterns on the SLM, imprinted onto the illuminating beam at a wavelength of 1030 nm. The images show the clockwise-rotating in-plane component of the plasmonic vector field, obtained in real-time by NNOM with a left-handed circularly polarized pump beam (clockwise-rotating field)

Figure 4 shows the real-time mapping of the in-plane near-field patterns carrying higher angular momenta, generated solely by applying additional azimuthal phase gradients with the SLM. We demonstrate seamless switching between these distinct modes, ranging up to *m* = 6, shown in Fig. 4a–f and in the supplementary movie S4. To the best of our knowledge, this is the first time such switching has been performed via an SLM, without constraints from the grating geometry (see examples in refs. ^26–32^).

**Fig. 5:**
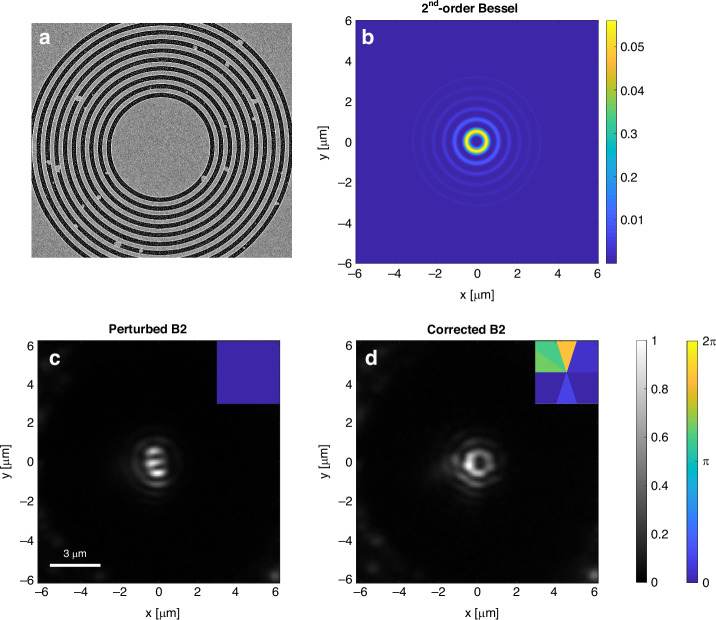
Correction of nanophotonic patterns using real-time nearfield microscopy as a feedback to wavefront shaping. **a** Au film patterned with coupling grating containing random structural defects. **b** calculated plasmonic pattern expected from the experimental conditions with no phase correction—2nd order plasmonic Bessel beam. **c** The measured pattern is severely distorted owing to the structural defects. The inset shows the uniform phase imprinted on the SLM, i.e., no phase correction. **d** The corrected plasmonic pattern after the phase closed-loop phase correction achieved using the real-time acquisition of the near-field pattern. The inset shows the phase pattern imprinted on the SLM

Finally, we demonstrate how the combination of wave-front shaping and real-time near-field imaging can be used to correct a nanophotonic pattern that was corrupted either by misalignment of the generating beam or structural defects in the coupler or in the surface. To increase the flexibility in the wave-front shaping, we used a conical lens to generate the ring shape, encoded the azimuthal phase information on that ring and measured plasmonic Bessel modes generated via coupling by a non-perfect grating. The damaged plasmonic system is shown in Fig. 5a and the modified setup is given in the supplementary.

Figure 5b depicts the distorted pattern caused by the structural defects in the coupling grating. Showing near-field measurement of the counter clockwise-rotating in-plane component that should correspond to 2nd-order Bessel beam without any phase encoding. Evidently, without any phase correction the pattern is severely distorted. Since the exact influence of the structural defects on the pattern distortion are unknown, the correction of the beam by wavefront shaping requires a real-time feedback that allows an iterative process to compensate for the distortion. By using a feedback loop involving the real-time monitoring of the near-field, we are able to correct the beam distortion and retrieve the 2nd-order Bessel shape (Fig. 5d). We show similar correction of 3rd order Bessel beam in Fig. S5 in the supplementary. The iterative process is elaborated in the supplementary section and in supplementary movies S5 and S6.

## Discussion

In summary, we demonstrated active shaping of nanophotonic fields, monitored and controlled by a direct feedback mechanism, opening the door to a wide range of new applications. This technology can now facilitate, for example, trapping and manipulation of nanoparticles as in optical tweezers, with order of magnitude improvement in its resolution and degree of control. Similarly, it can find various uses for integrated-circuits communication and computation such as homogenizing inputs to different waveguides and controlled excitation of emitters integrated in the photonic circuits.

This approach not only has the capability to create unique near-field patterns but also has the potential to correct and compensate for flaws and phase disorders caused e.g., during fabrication or by scatterers in the beam path, ultimately enabling the precise generation of complex wave functions, e.g., higher-order angular momentum near-field modes for future quantum computation and communication applications.

Furthermore, the implementation of advanced algorithms will facilitate the creation of even more intricate near-field wavefronts. This will promote the utilization of systems with several spatial and/or frequency modes^46^, allowing for even greater versatility in applications and further expanding the capabilities of nanophotonics. The development of more complex and adaptable near-field wavefronts holds the potential to revolutionize various scientific and technological domains, making our research a pivotal advancement in this exciting field.

## Materials and methods

### Experimental setup details

The pulsed laser utilized in our experiments is a mode-locked Ti:sapphire laser, specifically the Chameleon Ultra II, which delivers 140 fs pulses at a repetition rate of 80 MHz and a total output power of 3.7 Watts.

The laser beam is divided into two paths using a polarizing beam splitter in conjunction with a half-wave plate. The primary path, referred to as the pump beam, carries an average power of ~300 mW and is directed towards the sample. The secondary path, known as the OPO beam, is converted using an optical parametric oscillator (Chameleon OPO) to a wavelength of 1030 nm, with an average power of around 100 mW. This OPO beam is subsequently directed to a phase-only spatial light modulator (SLM, Holoeye PLUTO-2.1-NIR-015) before reaching the sample, where it is employed for surface plasmon excitation.

To optimize the beam profile on the sample, the pump beam is passed through a Variable Beam Expander (Broadband NIR, 750–1100 nm, 2X - 8X), which reduces the beam diameter before the objective. Consequently, the beam diameter on the sample is increased. The objective used for focusing the pump beam and imaging the nonlinear pattern is a 100 × Nikon LU Plan Fluor, with a numerical aperture (NA) of 0.9 and a working distance of 1 mm.

The OPO beam, after being reflected from the SLM, is directed to the objective (Mitutoyo, Infinity Corrected Objective, X20, NA 0.28) and then onto the sample. In the latter part of our work, involving wavefront shaping and feedback correction, we utilized double axicons (AX2510-B with a physical angle of 10°) and an imaging lens (THORLABS LB1409-B with a focal length of 1000 mm), as detailed in the experimental setup schematic (Fig. S.3).

Additionally, both the pump and OPO beams pass through λ/2, polarizer, and λ/4 plates to achieve the desired polarization.

The nonlinear signal measurements, depicted in the images, were captured using the iXon Ultra 888 EMCCD camera from Andor-Oxford Instruments.

### Sample preparation and fabrication

The sample consists of a gold layer with a thickness of 180 nm, deposited on a 1 mm thick glass coverslip using electron gun evaporation (Evatec). A 3 nm titanium adhesion layer was interposed between the gold and the glass. The coupling gratings’ geometries were fabricated using a Focused Ion Beam (FEI Helios NanoLab DualBeam G3 UC).

## Supplementary information

Movie S1 - Translation of nanophotonic focal point

Movie S2 - Fine tuning of nanophotonic focal point

Movie S3 - Focus Rotating

Movie S4 - OAM switching

Movie S5 - Correction of Bessel 2

Movie S6 - Correction of Bessel 3

Supplementary
