# Supplementary material for: Dynamic control and manipulation of near-fields using direct feedback
Source: Light Sci Appl. 2024 Oct 24;13:298. doi: 10.1038/s41377-024-01610-2 (PMC11499598; doi:10.1038/s41377-024-01610-2)
Supplement: Supplementary file 7 — Supplementary [file 41377_2024_1610_MOESM7_ESM.docx]

**Supplementary Information for**

**Dynamic Control and Manipulation of Near-fields Using Direct Feedback**

Jacob Kher-Aldeen^1^, Kobi Cohen^1^, Stav Lotan^1^, Kobi Frischwasser^1^, Bergin Gjonaj^2, 3^, Shai Tsesses^1, 4^, Guy Bartal^1^*.

^1^Andrew & Erna Viterbi Faculty of Electrical & Computer Engineering, Technion – Israel Institute of Technology, Haifa 3200003, Israel

^2^Department of Physical Engineering, Polytechnic University of Tirana - Faculty of Physical & Math Engineering, Tirana 1000, Albania

^3^Faculty of Medical Sciences, Albanian University, Durrës Street, Tirana 1000, Albania

^4^Department of Physics, MIT-Harvard Center for Ultracold Atoms and Research Laboratory of Electronics, Massachusetts Institute of Technology, Cambridge, MA, USA

*Corresponding author: [guy@ee.technion.ac.il](mailto:guy@ee.technion.ac.il)

**1. Far-field Wave-front shaping of near-field nanophotonic fields - simulations**

We calculate the relations between the pattern stored on the SLM and the resultant near-field pattern, obtained on the nanophotonic platform. The calculation consists of three stages: 1) Analytical expression of the phase pattern generated on the SLM. 2) Its Fourier transform, describing the shape of the beam incident on the sample. 3) Huygens-principle calculation of the near field pattern generated by the Incident beam on a ring-shaped coupler.

1.1 The wavefront shaping is performed by a spatial light modulator (SLM) placed in the Fourier plane of the grating coupler carved in a gold layer of about 160 nm thickness. In order to produce a ring-shaped beam at the coupler, which will match the coupler size, we create a radially-symmetric, radially-ascending phase on the SLM. This phase pattern is similar to that of a 0^th^-order Bessel beam whose Fourier transform generates the desired ring. The electric field of the wave reflected from the SLM is $E\left( r,\theta\right)=e^{-\pi\left( \frac{r}{r_{0}} \right)^{2}}e^{jar}$ (Figure S1a-b) where $r_{0}$ represents the size of the gaussian beam and $a$ is the radial rate of the phase advance that determines the ring size at the beam incidence on the grating. Adding an azimuthal ascent to the phase imprinted on the SLM results in a winding number $q$ corresponding to the number of times the phase completes 2π. The field pattern reflected from the SLM hence becomes $E\left( r,\theta\right)=e^{-\pi\left( \frac{r}{r_{0}} \right)^{2}}e^{j\left( ar+q\theta\right)}$ (Figure S1b-d).

After a sufficiently large propagation distance, the nearly-diffractionless Bessel-like component remains almost unchanged while the remainder, resulting from the gaussian-shape amplitude, undergoes a stronger diffraction and hence avoids the coupler entirely.

1.2 The resultant 0^th^ order Bessel beam goes through an objective lens, forming a ring shape on the objective exit. The field distribution of the ring can now be calculated and expressed by performing a Fourier transform on the beam incidenting the objective lens

$\mathcal{F}\left[ E\left( r,\theta\right) \right]=\frac{{r_{0}}^{2}}{4\pi}e^{-\frac{1}{4\pi}\left( k_{r}r_{0} \right)^{2}}e^{jqk_{\theta}}*\delta\left( k_{r}-a \right)=\frac{{r_{0}}^{2}}{4\pi}e^{-\frac{1}{4\pi}\left( k_{r}-a \right)^{2}{r_{0}}^{2}}\cdot e^{jqk_{\theta}}.$ (Figure S1e-h)

1.3. A plane or ring-shaped wave incident on a ring-shaped coupler generates a surface-wave or guided-wave pattern that depends on the phase of the beam at any given point on the coupler. The pattern created by this excitation can be calculated using a Huygens-principle simulation, which divides the ring into a series of point sources and sums up all the Hankel functions generated by each of them [1]. In our demonstrations, we use a metal-air interface as the nanophotonic platform, which supports a vector-field solution of a single surface plasmon polariton mode. The three vector components can be represented as two rotating in-plane components and one out-of-plane component [2]:

$$\left( \begin{matrix} E_{\sigma_{-}}^{SPP} \\ E_{\sigma_{+}}^{SPP} \\ E_{z}^{SPP} \end{matrix} \right)=\left( \begin{matrix} \frac{E_{x}^{SPP}+iE_{y}^{SPP}}{\sqrt{2}} \\ \frac{E_{x}^{SPP}-iE_{y}^{SPP}}{\sqrt{2}} \\ E_{z}^{SPP} \end{matrix} \right)\propto\left( \begin{matrix} J_{q-2}\left( k_{SPP}\rho\right)e^{i\left( q-2 \right)\theta} \\ J_{q}\left( k_{SPP}\rho\right)e^{iq\theta} \\ J_{q-1}\left( k_{SPP}\rho\right)e^{i\left( q-1 \right)\theta} \end{matrix} \right)e^{-\left| k_{z} \right|z}$$

Where the in-plane field is expressed via its rotating field components $E_{\sigma_{+}}^{SPP}=E_{x}^{SPP}+jE_{y}^{SPP}$ and $E_{\sigma_{-}}^{SPP}=E_{x}^{SPP}-jE_{y}^{SPP}$ [2]. Illuminating the sample with a $\hat{\sigma}_{+}$ polarized pump recovers the shape of the $\hat{\sigma}_{+}$ component of the plasmonic vector field at the interface, i.e., $J_{q}\left( k_{SPP}\rho\right)e^{iq\theta}$. Figure S1 shows a simulation of all stages (1-3) of such nanophotonic near-field pattern whose angular momentum is generated and controlled by phase-front shaping.


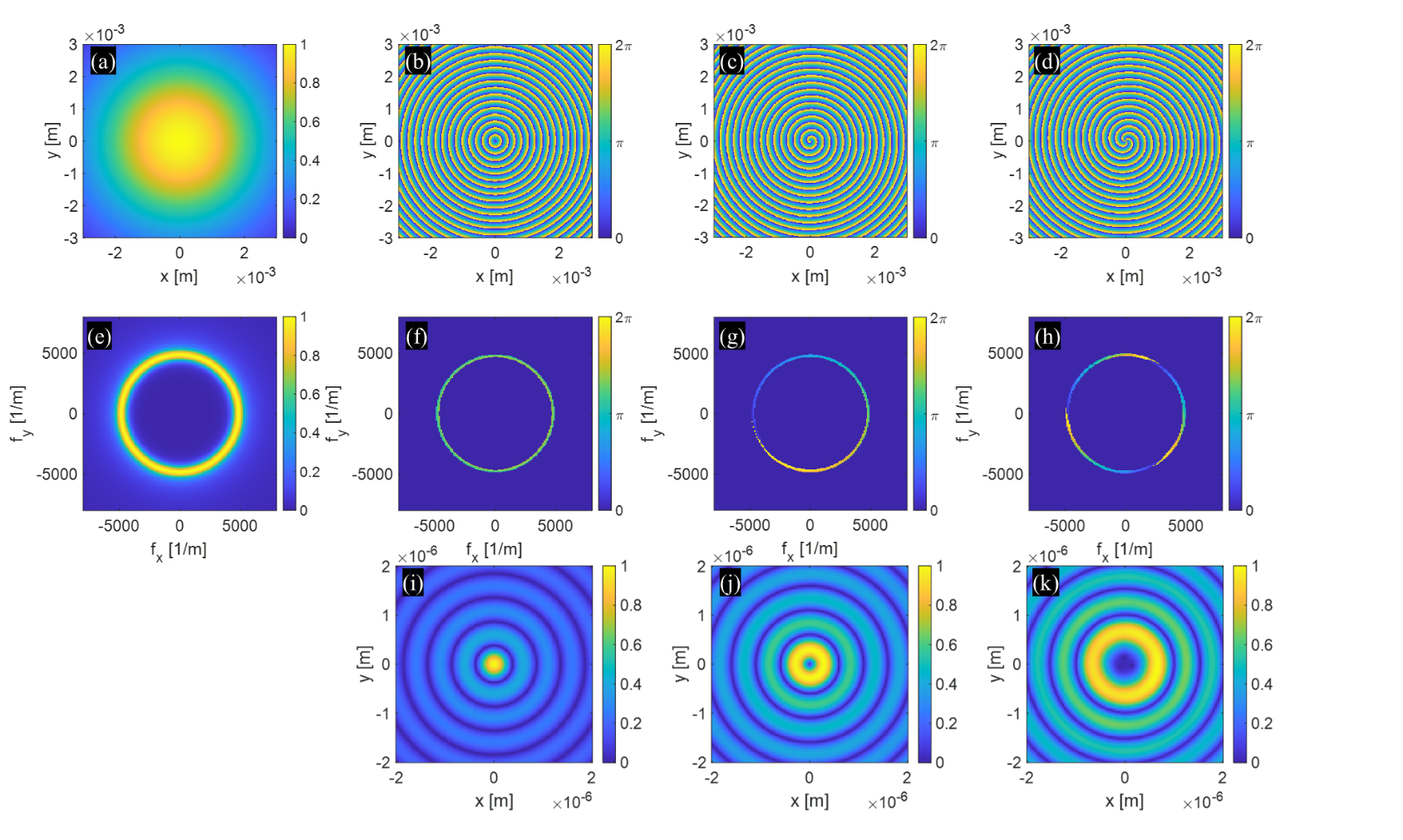


**Fig. S1.** (a-d) Phase-shaped beam reflected from the SLM: Amplitude (a) and different phases with topological charge $q=1,2,3$, respectively (b-d). (e) Amplitude and (f-h) phases of the same beams after passing through an objective lens. (i-k) Huygens-principle simulation of the resulting beam on the nanophotonic platform.

**2. Control over nanophotonic focal point**

The same phase control can be used for steering a plasmonic focal point, represented in a two-dimensional platform, as a 0-th order Bessel function. This can be done by merely translating the phase pattern on the SLM by a number of pixels. The translation of nanophotonic focal spot, shown in Figure 2 in the manuscript and in video S1, consists of 1.43 µm shift corresponding to translation of 50 pixels on the SLM. This provides a potential resolution of sub 30 nm in controlling the location of the focal spot as shown in video S2. Figure S2 shows a simulation of all stages (1-3) of such control over a nanophotonic focal spot by far-field wavefront shaping, at the same form as Figure S1.


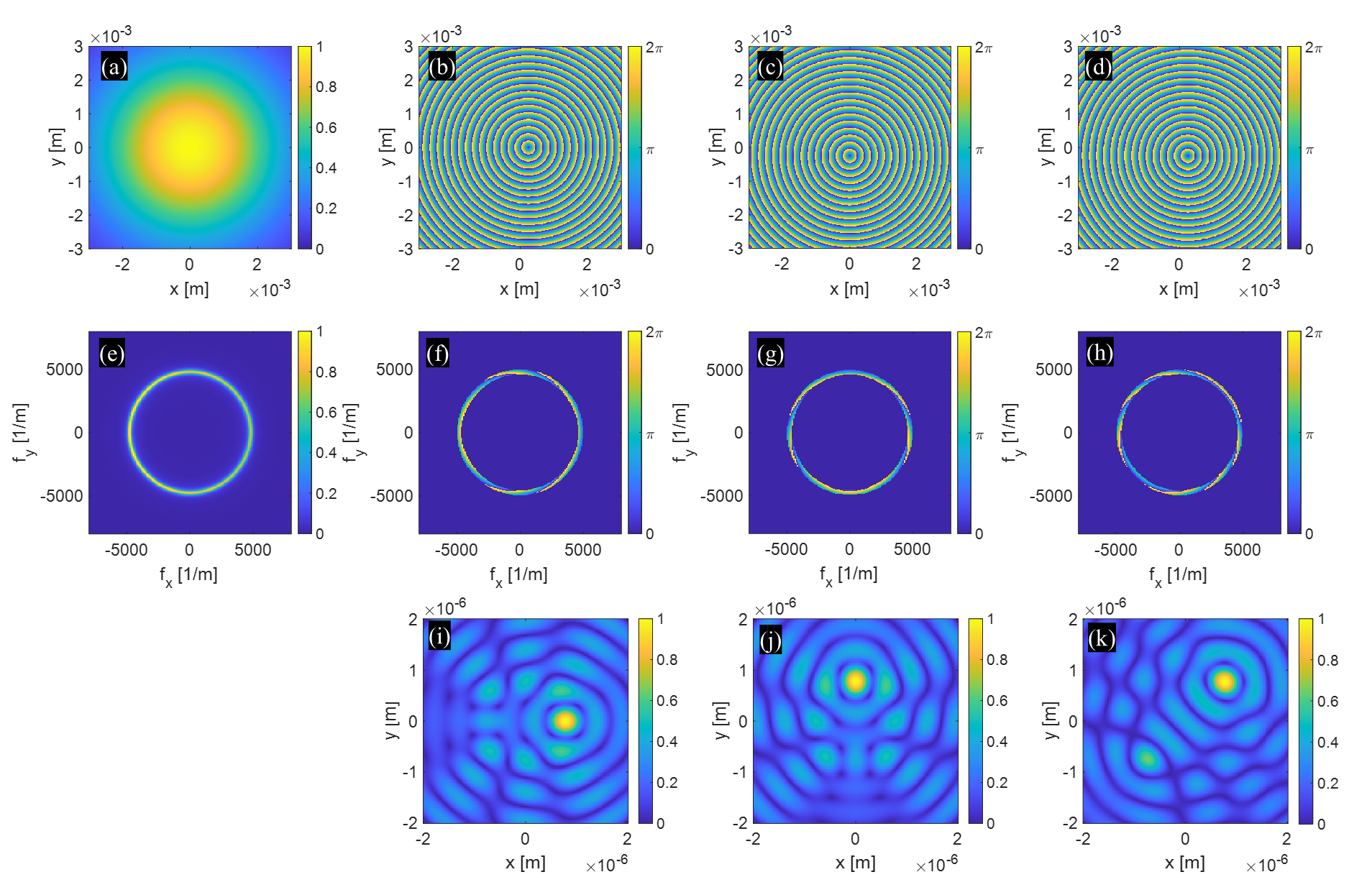


**Fig. S2.** (a-d) Phase-shaped beam reflected from the SLM: Amplitude (a) and three different translations of the phase pattern (b-d). (e) Amplitude and (f-h) Phases of the same beams after passing through an objective lens. (i-k) Huygens-principle simulation of the resulting beam on the nanophotonic platform.

**3. Correction of nanophotonic wave patterns using iterative feedback loop**

We show here the modified optical system that was set to support the correction of nanophotonic patterns by phase engineering of the incident beam.


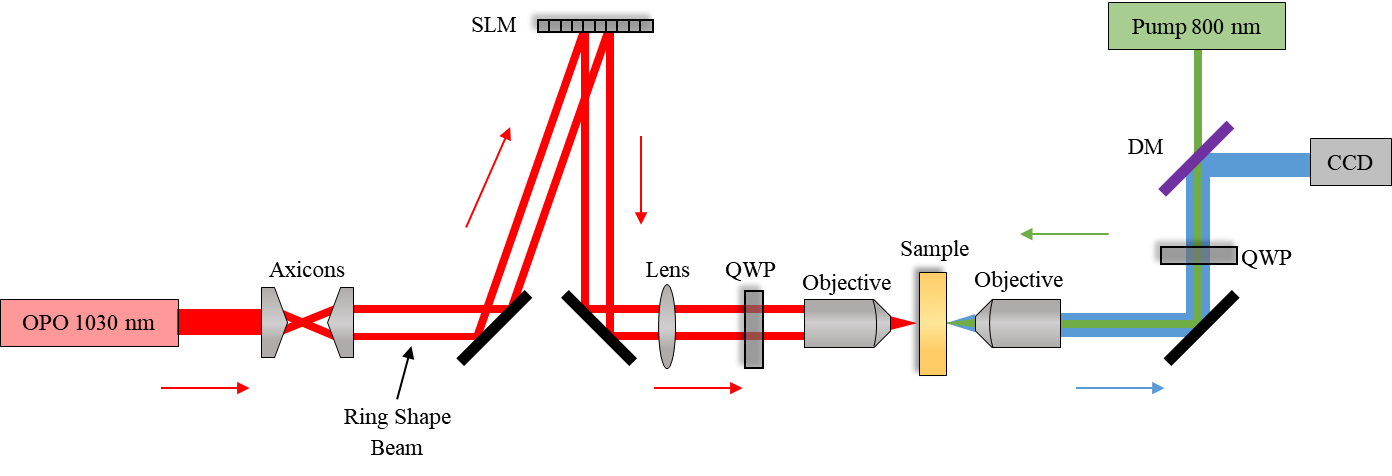


**Fig. S3. Correction of nanophotonic wave patterns using feedback-assisted wavefront shaping).** The axicons added form a ring on the SLM, saving the need for the radial phase patterning and allowing direct imaging of the SLM onto the coupling grating*.*


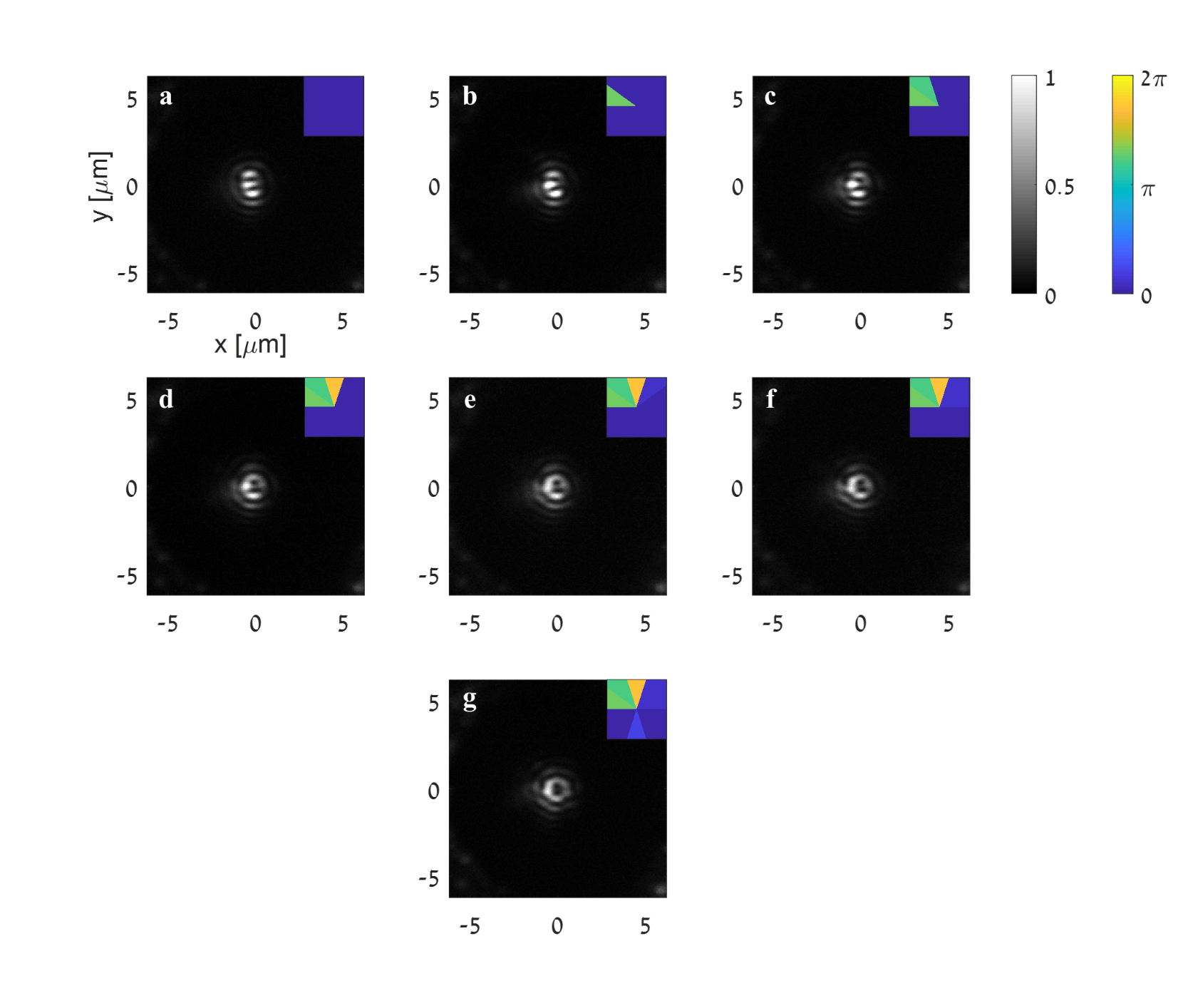


**Fig. S4. The iterative process to correct a 2^nd^-order plasmonic Bessel beam using feedback-assisted wavefront shaping.**


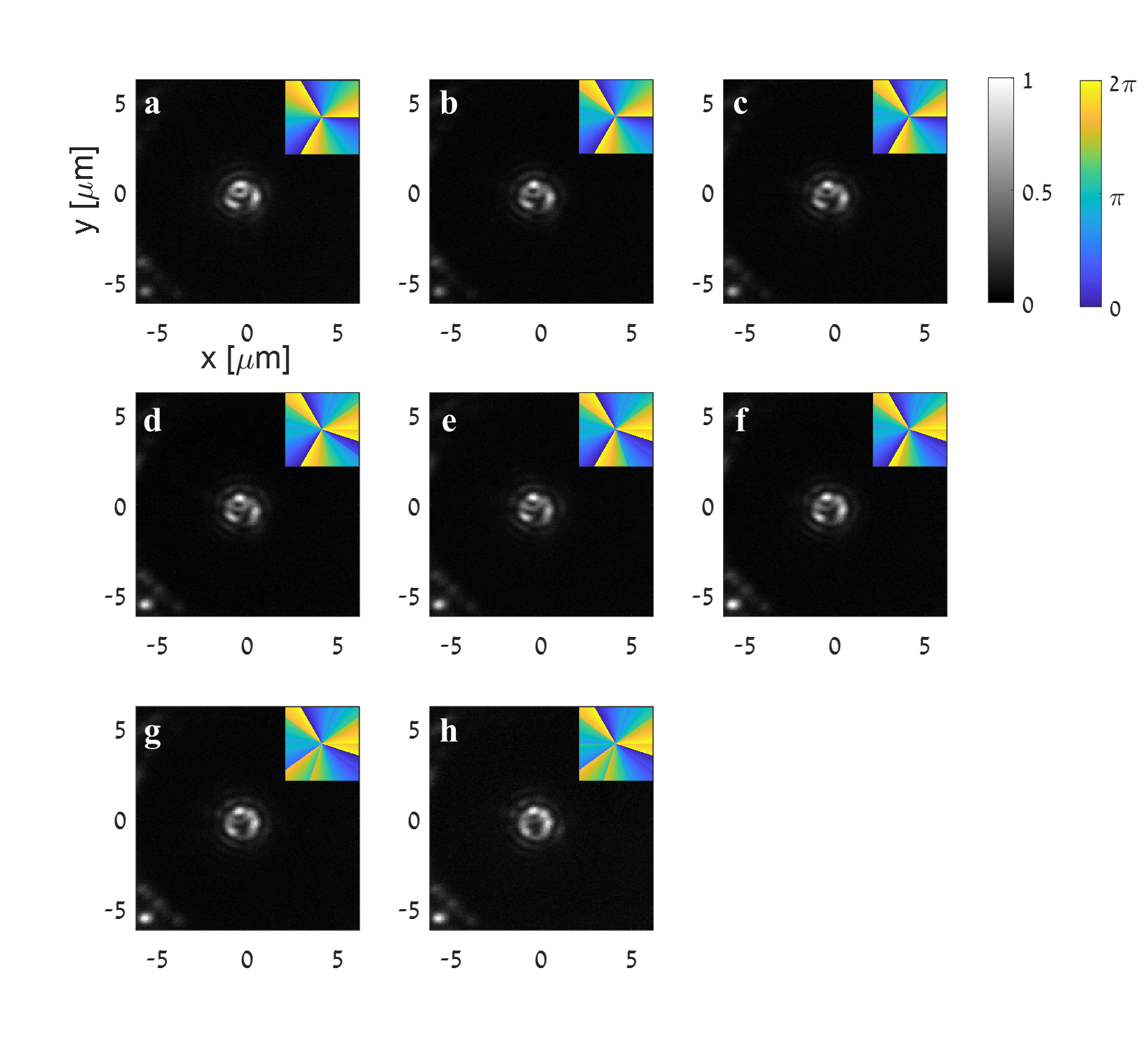


**Fig. S5. The iterative process to correct a 3^nd^-order plasmonic Bessel beam using feedback-assisted wavefront shaping.**

**3. Sample damage threshold and decay length of the plasmonic patterns**

While the pump laser is intensive and can potentially damage the sample, the short pulses (~140FS) reduces significantly the risk for such a damage. We have already successfully implemented this approach both on gold surface and Silicon waveguides. In particular, we have experimentally assessed the damage threshold of the plasmonic samples we used and found it to be twice as much as we need.

The detailed parameters we use are appended below:

| Pulse duration | $140 fs$ |
| --- | --- |
| Laser power | $100 mW$ (before obj.) |
| Pulse repetition | $80 MHz$ |
| Pulse energy | $\frac{100\cdot{10}^{-3}}{80\cdot{10}^{6}}=1.25\cdot{10}^{-9} J$ |
| Pulse power | $\frac{1.25\cdot{10}^{-9}}{140\cdot{10}^{-15}}=8.9\cdot{10}^{3} W$ |
| Beam diameter on sample | $\sim4 \mu m$ |
| Beam area on sample | $12.6\cdot{10}^{-12} m^{2}$ |
| Pulse Intensity | $\frac{8.9\cdot{10}^{3}}{12.6\cdot{10}^{-12}}=709\cdot{10}^{12} Wm^{-2}$ |
| Sample damage threshold | $\sim200 mW \to1419\cdot{10}^{12} Wm^{-2}$ |

The pump illuminates the pattern area at normal incidence so its decay into the metal

Can be calculated as follows:

$$k_{z}=\sqrt{\varepsilon_{m}}k_{0}=\left( 1.21+38.5i \right)\cdot{10}^{6} m^{-1}$$

$$L_{decay}=\frac{1}{Im\left\{ k_{z} \right\}}=26 nm$$

The pattern decay is the plasmonic decay length at 1030nm wavelength which is:

$$k_{SPP}=\left( 6.17+0.0051i \right)\cdot{10}^{6} m^{-1}$$

$$L_{decay}=\frac{1}{Im\left\{ k_{SPP} \right\}}=200 \mu m$$

Hence the decay of the plasmonic pattern is much larger than the much larger than the pattern size.

**References:**

1. T. V. Teperik, A. Archambault, J. J. Greffet, and F. Marquier, "Huygens-Fresnel principle for surface plasmons," Opt. Express, Vol. 17, Issue 20, pp. 17483-17490 **17**, 17483–17490 (2009).
2. K. Frischwasser, K. Cohen, J. Kher-Alden, S. Dolev, S. Tsesses, and G. Bartal, "Real-time sub-wavelength imaging of surface waves with nonlinear near-field optical microscopy," Nat. Photonics 2021 156 **15**, 442–448 (2021).
